# Evidence of the Amplitude of Accommodation of School-Going Children in the 21^st^ Century

**DOI:** 10.22599/bioj.303

**Published:** 2023-06-20

**Authors:** Alvin Jeffrey Munsamy, Andile Ngema, Seyuri Bisetty, S’fundo Lushaba, Nomvelo Mayaba, Bongakonke Mthiyane, Nombuso Nyathi, Amen Thabethe

**Affiliations:** 1 University of KwaZulu-Natal, ZA

**Keywords:** accommodation, schoolchildren, Hofstetter, amplitude of accommodation, push-up method, myopia

## Abstract

**Purpose:**

The study sought to compare the normative amplitude of accommodation (AoA) in school-going children from studies in the 21st century, based on pooled estimates from meta-analyses, to assess their agreement to Hofstetter’s average formula.

**Methods:**

A PRISMA checklist was used to conduct the review. PubMed, EBSCOHOST and Medline electronic databases were employed, and hand searching resulting in 259 studies up to July 2021. After title and abstract screening, 12 studies underwent full-text screening, resulting in five studies for data extraction. The pooled effect size was determined using meta-analyses for sub-groups by age. A one-sample t-test was used to compare the pool-effect size estimates (monocular) to the expected AoA from Hofstetter’s average formula.

**Results:**

The comparison of pool estimates of AoA with the expected Hofstetter’s average formula for the age sub-groups showed significant mean differences for: six-year olds: mean difference of –3.4 D (95% CI: –5.85; –1.04; p = 0.025); nine-year olds: mean difference of –4.1D (95% CI: –7.95; –0.20; p = 0.043); ten-year olds: mean difference of –4.6D (95% CI: –8.57; –0.54; p = 0.035) and 11-year olds: mean difference of –5.2 D (95% CI: –8.06; –2.40; p = 0.005). According to the quality assessment tool used, overall, the body of evidence was of good quality.

**Conclusion:**

Hofstetter’s prediction of normative amplitude of accommodation today may over-estimate for children aged six, nine, 10 and 11. The observed under-accommodation estimates from these comparisons may warrant consideration in assessing for a larger lag of accommodation in these age groups with myopia or pre-myopia, as part of the surveillance for progression.

## Introduction

The amplitude of accommodation (AoA) represents the maximal accommodative level, or closest near focusing response, that can be produced with maximal voluntary effort in the fully corrected eye (Ciuffreda 2006). It is incorporated in the routine eye and vision examination (Evans 2007). Hofstetter established the predicted AoA formula that is used to measure the AoA by using Donder’s push-up method, established in the 1940s. The average amplitude of accommodation, in dioptres, for a child of a given age was estimated by Hofstetter to be 18.5 – (0.30* patient age in years) (Hofstetter 1950).

Recent reports question Hofstetter’s formula and that poor accommodation in children might be relevant for increasing levels of accommodation symptoms and possibly myopia progression. The twenty first century society has become more accustomed to working indoors, at distances closer than the normal reach; which may impact the typical functioning of the visual system and, more specifically, the accommodative system of the human eye. Accounting for the ergonomic impacts of close work today, the relevance of addressing changing accommodative abilities as a result of the constant demands of close work may call for accurate reference norms for AoA.

Evidence (Duclos 1989) from as far back as 1989 shows a difference between Hofstetter’s predictions and the clinical presentation, for ages between 10 to 29 years, which fell outside the two-standard-deviation rule. Collating studies on vulnerable populations, such as children from the 21st century, and comparing this data to the gold standard for AoA measurements, as determined by Hofstetter, may be valuable.

This study aims to compare normative AoA in school-going children from studies in the 21st century, based on the pooled estimates from meta-analyses, to assess their agreement with Hofstetter’s average formula. This will determine if there is an under- or over-estimation by Hofstetter’s average prediction in school-going children living in the 21st century.

### Materials and Methods

This systematics review was guided using the Joana Briggs Institute Reviewers’ Manual (Joanna Briggs Institute 2015).The PRISMA flow from the Joanna Briggs Institute Reviewers’ Manual (Moher et al. 2009) was used to conduct this systematic review. The PICO [*Population* (school children; both genders; within the 2001 to 2021 timeframe; not restricted to South Africa), *Intervention* (normal clinical amplitude of accommodation), *Control/Comparison* (Hofstetter’s average equation based on the outcomes of this study), and *Outcomes* (agree or disagree with Hofstetter’s average equation based on the outcomes of this study)] framework was utilised to formulate the research question.

### Study search and selection

Three databases were utilised to source relevant studies viz. PubMed, EBSCOHOST (academic search complete), and Medline. The search only included those studies published from 2001 up to 2021, using the following terms: ‘amplitude’, ‘accommodation’, ‘children’, and ‘school’, with all possible combinations. Title screening commenced by two members (AN and NM) of the team, guided by the PICO framework (‘schoolchildren’, ‘amplitude of accommodation’). All accepted titles went through an independent abstract screening, which was completed by two reviewers (NN and SB) from the team using an abstract screening tool. All abstracts that met the selection criteria at that stage were then subjected to full-text screening, which involved two team members (NM and SB). Articles that failed to meet the study criteria were excluded, with reason before undergoing data extraction.

### Inclusion and exclusion criteria

All studies that sought to determine the amplitude of accommodation using the push-up method in children, in keeping with Donder’s approach, were included. Studies only on normal children without accommodative anomalies were included. All studies, irrespective of study design, from 2001 to 2021 were included and were not limited to geographic location or language of publication. Studies had to be available with the full text, any additional studies that were applicable and were not sourced from the database searches were also included.

Studies with populations older than 18 years, with AoA measurements for age ranges instead of individual ages were excluded. Studies that measured only binocular AoA, used clinical populations with accommodation and binocular vision dysfunctions were excluded. All published before 2001 as well as non-peer-reviewed grey literature (source that does not traditionally publish) were also excluded.

### Data extraction

Data were extracted by two members (SB and NM), who retrieved all relevant data from the included studies. The following information was extracted from all relevant studies using a table: first author; year of publication; study setting; title; the aim of the study; the objective of the study; sample size; number of children tested; age group; year of study; and study outcomes: mean amplitude of accommodation (monocular) per age group.

### Risk-of-bias assessment

The quality of the included studies was assessed using the Quality Assessment Tool for Observational Cohort and Cross-Sectional Studies to provide a complete quality assessment. The tool has 14 questions for the publication that address all the issues that have a bearing on the quality of evidence (Shuang et al. 2014).

### Data Analysis

Data analysis was conducted using Stata 16, which facilitated the calculation of the pooled estimates. As a result, tables and forest plot(s) of within-group pooled estimates, and confidence levels of amplitude of accommodation for relevant age sub-groups, were produced. A one-sample t-test was used to compare the within-group pool estimates for each sub-group by age with the expected AoA from Hofstetter’s average formula. The criterion for statistical significance was set at 5%.

## Results

### Systematic search results

**Figure 1:**
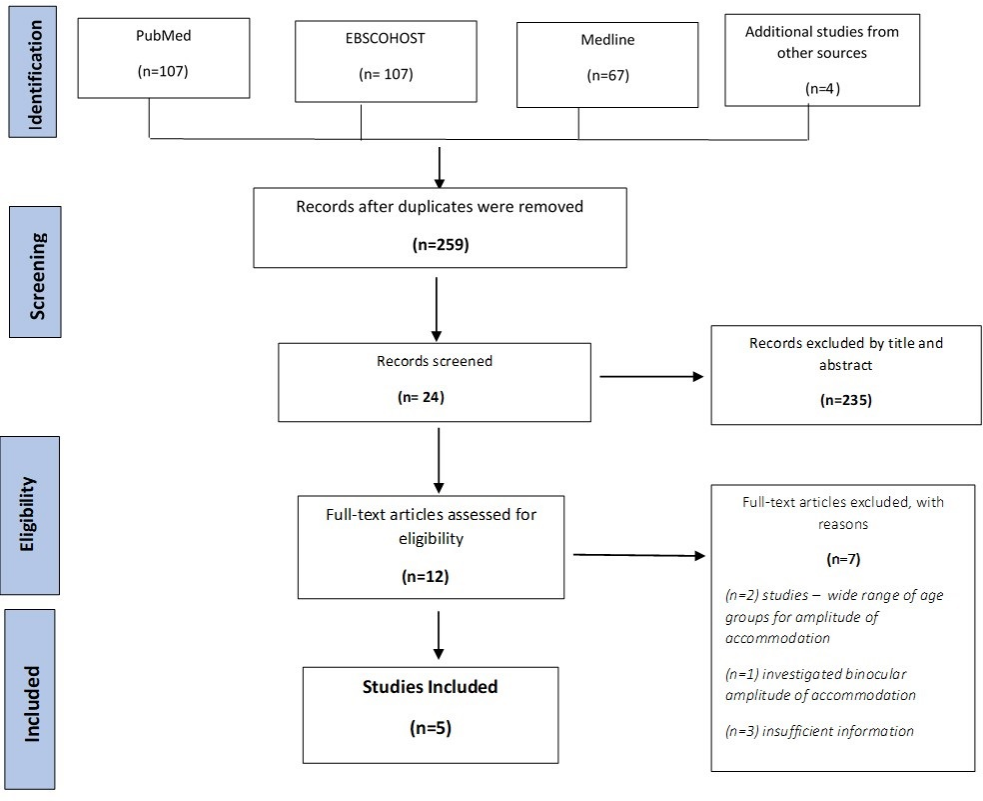
Prisma Flow chart for article selection process.

The electronic search yielded 283 studies, and a manual search through the reference lists of previous review studies yielded four more papers. After duplicate removal, 259 studies met the inclusion criteria. Irrelevant studies were then removed, resulting in a total of 235 studies being eliminated after the title and abstract screening using the selection criteria. The remaining 24 abstracts were screened systematically, resulting in 12 studies qualifying for full-text screening, while seven studies were excluded for various reasons (see Figure 1). Finally, five studies were used for the data extraction. The Prisma flow chart for study selection is shown in Figure 1.

### Study characteristics

Table 1 illustrates the characteristics of the studies that were used for meta-analysis, namely: author; the year in which the study was published; geographical information, which included the continent as well as the country; overall sample size; age groups with the corresponding sample sizes; and lastly, the AoA measurement in dioptres, measured using the push-up technique from the right eye. These studies were carried out between 2008 and 2018 and included a study population aged between six and 15 years. Articles were gathered from Europe, Africa, Asia, South America and North America. All studies were quantitative and cross-sectional in design, with an overall sample size from the included studies of 6,276.

**Table 1:** Study characteristics and patient population of studies included in the meta-analysis.


AUTHOR	YEAR	CONTINENT	COUNTRY	N	AGE (YEARS)	n	AOA (D)* MEAN ± SD**

**Ovenseri-Ogbomo et al**. (Ovenseri-Ogbomo et al. 2012)	2012	Africa	Ghana	435	8	52	19.0 ± 2.1

					9	71	18.4 ± 2.5

					10	45	17.1 ± 2.5

					11	69	16.1 ± 3.2

					12	69	16.3 ± 2.8

					13	67	16.1± 3.4

					14	62	15.5 ± 2.8

**Sergienko et al**. (Sergienko and Nikonenko 2015)	2015	Europe	Ukraine	155	8	9	10.7 ± 1.5

					9	7	10.6 ± 1.8

					10	4	9.3 ± 0.5

					11	9	9.9 ± 1.5

					12	9	10.1 ± 1.2

					13	15	9.3 ± 1.0

					14	30	8.9 ± 1.6

					15	24	8.9 ± 1.2

**Castagno et al**. (Castagno et al. 2017)	2017	South America	Brazil	867	6	55	15.9± 2.9

					7	74	16.2 ± 2.9

					8	99	15.1 ± 2.9

					9	96	16.3 ± 4.2

					10	99	16.8± 4.5

					11	103	14.9 ± 3.1

					12	93	15.3 ± 3.9

					13	108	14.2 ± 2.7

					14	90	13.9 ± 2.7

					15	32	13.3 ± 2.4

**Hashemi et al**. (Hashemi et al. 2018)	2018	Asia	Iran	5444	6	214	15.3± 0.4

					7	810	15.0 ± 0.2

					8	981	14.8 ± 0.2

					9	1035	14.5 ± 0.2

					10	872	14.1 ± 0.2

					11	913	14.0 ± 0.2

**Benzoni et al**. (Benzoni and Rosenfield 2012)	2007	North America	USA	60	5	6	16.2 ± 1.7

					6	14	14.0 ± 0.8

					7	10	10.9 ± 0.8

					8	12	12.2 ± 1.0

					9	9	12.3 ± 1.0

					10	9	12.4 ± 1.4


**AoA:** Amplitude of accommodation, *: Measurements using the push-up technique; **: Measurements from the right eye.

Table 2 illustrates the studies that were grouped according to age, ranging from six to 15 years. The characteristics of these studies include age group; author; the year in which the study was published; the sample sizes of the age groups for each study; and the AoA measurement in dioptres measured using the push-up technique from the right eyes.

**Table 2:** Sub-groups by ages from identified studies used in the meta-analysis. Hofstetter’s expected amplitude of accommodation (AoA) for ages 6–15 years, compared with expected values; according to Hofstetter’s average equation: expected amplitude 18.5–0.3 (age), Minimum: 15–0.25 (age), Maximum: 21–0.4 (age).


AGE(YEARS)	STUDY (AUTHORS)	YEAR	N	AMPLITUDE OF ACCOMMO-DATION ± SD	EXPECTED AVERAGE AOA IN DIOPTRES (MIN, MAX)	POOLED ESTIMATE(95% CI)	MEAN DIFFERENCE OF POOLED ESTIMATE VS. EXPECTED AOA	95%CI	P-VALUE

**6**	Castagno et al.	2017	55	15.9 ± 2.9	16.70 (13.5, 22.60)	14.91 (13.85, 15.96)	–3.44	–5.85; –1.04	**0.025**

	Hashemi et al.	2018	214	15.3 ± 0.4					

	Benzoni et al.	2007	14	14.0 ± 0.8					

**7**	Castagno et al.	2017	74	16.2 ± 2.9	16.40 (13.25, 22.20)	13.62 (10.41, 16.83)	–4.47	–11.37; 2.44	0.108

	Hashemi et al.	2018	810	15.0 ± 0.2					

	Benzoni et al.	2007	10	10.9 ± 0.8					

**8**	Ovensari-Ogbomo et al.	2012	52	19.0 ± 2.1	16.10 (13, 21.80)	14.11 (11.32, 16.89)	–3.92	–8.01; 0.18	0.057

	Sergienko et al.	2015	9	10.7 ± 1.5					

	Castagno et al.	2017	96	16.3 ± 4.2					

	Hashemi et al.	2018	981	14.8 ± 0.2					

	Benzoni et al.	2007	12	12.2 ± 1.0					

**9**	Ovensari-Ogbomo et al.	2012	71	18.4 ± 2.5	15.80 (12.75, 21.40)	13.80 (11.51, 16.09)	–4.08	–7.95; –0.20	**0.043**

	Sergienko et al.	2015	7	10.6 ± 1.8					

	Castagno et al.	2017	96	16.3 ± 4.2					

	Hashemi et al.	2018	1035	14.5 ± 0.2					

	Benzoni et al.	2007	9	12.3 ± 1.0					

**10**	Ovensari-Ogbomo et al.	2012	45	17.1 ± 2.5	15.50 (12.50, 21.00)	13.10 (10.26, 15.94)	–4.55	–8.57; –0.54	**0.035**

	Sergienko et al.	2015	4	9.3 ± 0.5					

	Castagno et al.	2017	99	16.8 ± 4.5					

	Hashemi et al.	2018	827	14.1 ± 0.2					

	Benzoni et al.	2007	9	12.4 ± 1.4					

**11**	Ovensari-Ogbomo et al.	2012	69	16.08 ± 3.19	15.20 (12.25, 20.60)	12.69 (10.52, 14.86)	–5.23	–8.06; –2.40	**0.005**

	Sergienko et al.	2015	9	9.9 ± 1.6					

	Castagno et al.	2017	103	14.9 ± 3.1					

	Hashemi et al.	2018	913	14.0 ± 0.2					

**12**	Ovensari-Ogbomo et al.	2012	69	16.3 ± 2.8	14.90 (12, 20.20)	13.11 (8.65, 17.56)	–4.59	–12.83; 3.65	0.139

	Sergienko et al et al.	2015	9	10.1 ± 1.2					

	Castagno et al.	2017	93	15.3 ± 3.9					

**13**	Ovenseri-Ogbomo et al.	2012	67	16.1 ± 3.4	14.60 (11.75, 19.80)	12.34 (8.01, 16.69)	–5.30	–13.99; 3.39	0.120

	Sergienko et al.	2015	15	9.3 ± 1.0					

	Castagno et al.	2017	108	14.2 ± 2.7					

**14**	Ovenseri-Ogbomo et al.	2012	62	15.5 ± 2.8	14.30 (11.50, 19,40)	12.25 (8.01, 16.49)	–5.75	–14.28; 2.79	0.101

	Sergienko et al.	2015	30	8.9 ± 1.6					

	Castagno et al.	2017	90	13.9 ± 2.7					

**15**	Sergienko et al.	2015	24	8.9 ± 1.2	14.00 (11.25, 19.00)	10.60 (6.39, 14.82)	–7.41	–35.42; 20.61	0.184

	Castagno et al.	2017	32	13.3 ± 2.4					


### Meta-analysis of amplitude of accommodation by age from relevant studies

**Figure 2:**
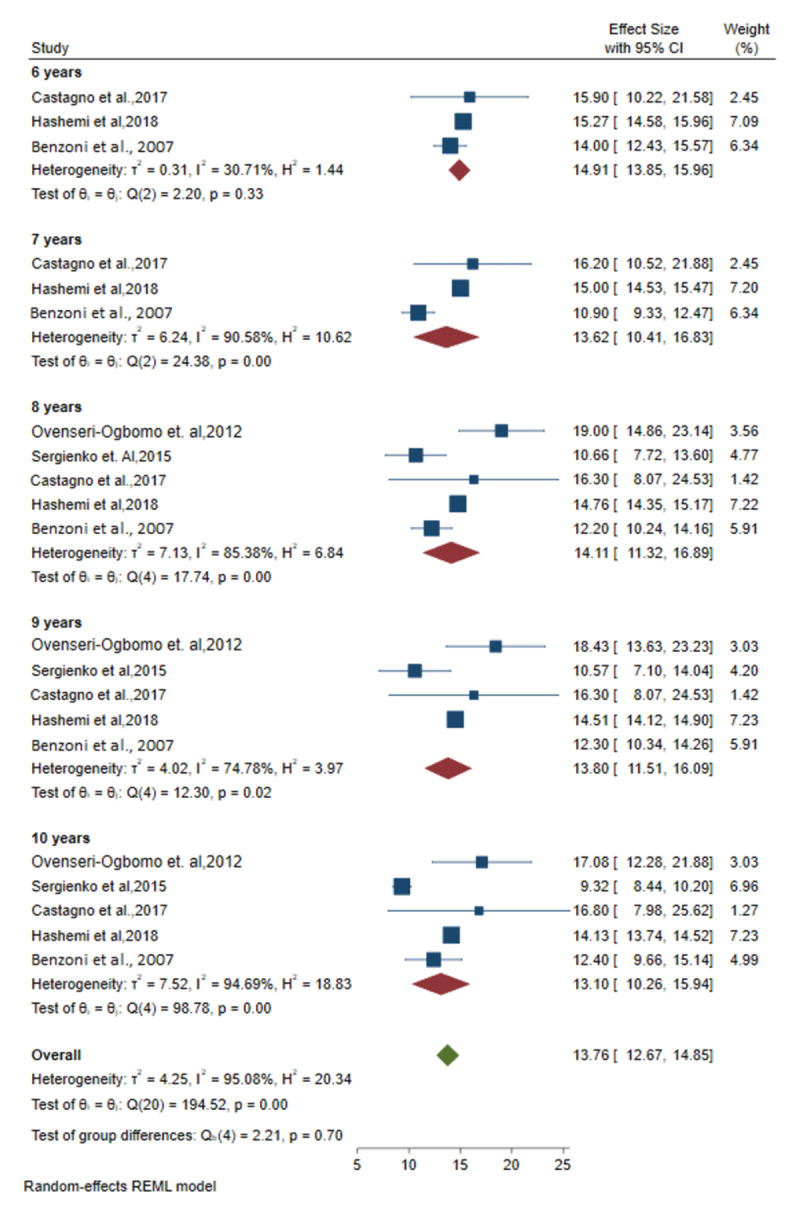
Forest plots showing the pooled outcomes of the amplitude of accommodation for included studies of age groups 6 to 10 years.

**Figure 3:**
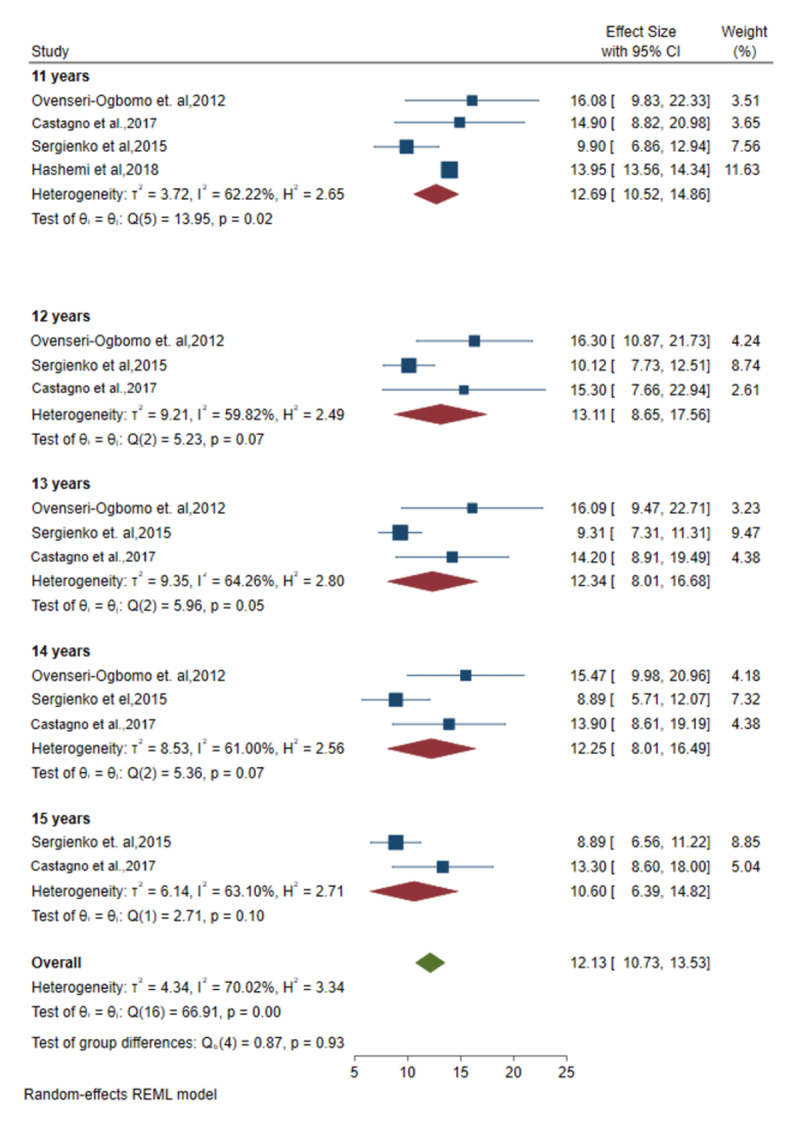
Forest plots of the pooled amplitude of accommodation for included studies of age groups 11 to 15 years.

The mixed effect meta-analysis model results are presented in Figure 2 and Figure 3. In these plots, the mean and standard deviations (SD) of the amplitude of accommodation are reported for each age group, from six to 10 years, as shown in Figure 2; and from 11 to 15 years, as shown in Figure 3. These statistics were reported by five studies and results were pooled and computed (Benzoni and Rosenfield 2012; Ovenseri-Ogbomo et al. 2012; Sergienko and Nikonenko 2015; Castagno et al. 2017; Hashemi et al. 2018).

In subgroup analysis (Figure 2), the pooled mean outcome for the amplitude of accommodation at age **six years** was found to be 14.9D (95% CI, 13.9 to 16; *I*^2^ = 30.7%) from three studies. The **seven-year-olds** had an outcome of 13.6D (95% CI, 10.4 to 16.8; *I*^2^ = 90.6%) from three studies. At age **eight years**, it was found to be 14.1D (95% CI, 11.3 to 16.9; *I*^2^ = 85.4%) from five studies. For **nine-year-olds** it was 13.8D (95% CI, 11.5 to 16.1; *I*^2^ = 74.8%) from five studies; and for **10-year-olds** it was 13.1D (95% CI, 10.2 to 15.9; *I*^2^ = 94.7%) from five studies.

In subgroup analysis (Figure 3) at **age 11**, the pooled mean outcome was 12.7D (95% CI, 10.5 to 14.9: *I*^2^ = 62.2%) from five studies. At **age 12**, it was found to be 13.1D (95% CI, 8.7 to 17.6; *I*^2^ = 59.9%) from three studies. At **13 years**, it was found to be 12.3D (95% CI, 8.0 to 16.7; *I*^2^ = 64.3%) from three studies**. At 14 years**, it was 12.3D (95% CI, 8.0 to 16.5; *I*^2^ = 61.0%) from three studies; and for the age group **15 years**, it was 10.6D (95% CI, 6.4 to 14.8; *I*^2^ = 63.1%) from two studies.

The study’s within-group heterogeneity was investigated in terms of population, age, and outcome measurements of the AoA. Statistical heterogeneity was assessed by examining the forest plot’s confidence intervals and using the *I^2^* statistics, which were assessed per age group. According to the results, the outcome of the amplitude of accommodation for six-year-old children showed low heterogeneity. Ages seven, eight, and 10 years showed high heterogeneity; while ages nine, and 11–15 showed moderate heterogeneity. An estimate of *I^2^* > 50% was considered as indicating substantial heterogeneity and *I^2^ >* 75% suggested considerable heterogeneity (Higgins, Thompson & Spiegelhalter 2009).

### Comparison of pooled estimates and Hofstetter’s expected amplitude of accommodation

Table 2 also illustrates Hofstetter’s expected amplitude of accommodation (AoA) for ages six to 15 years, in comparison to expected values. This was calculated using Hofstetter’s average equation: expected amplitude 18.5–0.3 (age), using the one-sample t-test. The sub-group meta-analyses show that the estimate of the mean pooled outcomes was statistically significant for age groups six, nine, 10, and 11 years.

The sub-group meta-analysis for the six-year-old group shows that the estimate of the pooled mean outcome for AoA was below Hofstetter’s expected average, with a mean difference of –3.44 D (95% CI: –5.85; –1.04; p = 0.025).

The sub-group meta-analysis for the nine-year-old group shows that the estimate of the pooled mean outcome for AoA was below Hofstetter’s expected average, with a mean difference of –4.08D (95% CI: –7.95; –0.20; p = 0.043).

The sub-group meta-analysis for the ten-year-old group shows that the estimate of the pooled mean outcome for AoA was below Hofstetter’s expected average, with a mean difference of –4.55 D (95% CI: –8.57; –0.54; p = 0.035).

The sub-group meta-analysis for the 11-year-old group shows that the estimate of the pooled mean outcome for AoA was below Hofstetter’s expected average, with a mean difference of –5.23 D (95% CI: –8.06; –2.40; p = 0.005).

**Figure 4:**
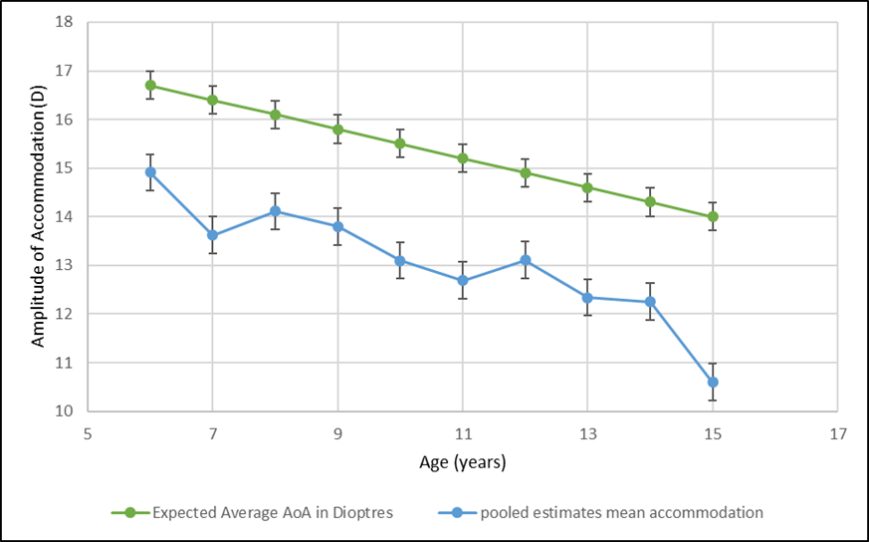
Amplitude of accommodation in diopters as a function of age from pooled estimates and Hofstetter’s expected mean accommodation.

Figure 4. shows the comparison of the pool-estimates of the mean amplitude of accommodation for each age group in relation to the predicted amplitude of accommodation calculated from Hofstetter’s average equation: 18.5–0.3(age). This graphical representation illustrates Hofstetter’s lack of agreement with studies from the twenty-first century.

### Risk of bias of included studies

The Quality Assessment Tool for Observational Cohort and Cross-Sectional Studies was used to assess the quality of the five studies. This tool comprises fourteen questions, with less than ‘7/14’ ‘yes’ responses indicating *poor* quality; ‘7–8/14’ ‘yes’ responses indicating *fair* quality; and ‘9–14/14’ ‘yes’ responses indicating *good* quality. In our study, some factors were not applicable and were therefore excluded from our rating; thus, all the studies used were rated out of 11. Study one rated ‘9/11’ ‘yes’ responses and was graded as good. Study two rated ‘10/11’ ‘yes’ responses and was graded as good. Study three rated ‘9/11’ ‘yes’ responses and was graded as good. Study four rated ‘7/11’ ‘yes’ responses and was graded as fair. Study five rated ‘9/11’ ‘yes’ responses and was graded as good. Overall, the quality of evidence was rated as good quality. Table 3 shows the appraisal scores of the studies.

**Table 3:** Results of the appraisal using the Quality Assessment Tool for Observational Cohort and Cross-sectional Studies (*Study Quality Assessment Tools | NHLBI, NIH*, no date).


FIRST AUTHOR AND YEAR	1	2	3	4	5	6	7	8	9	10	11	12	13	14

Ovenseri-Ogbomo et al. 2012 (Ovenseri-Ogbomo et al. 2012)	Y	Y	Y	Y	Y	N	N	N/A	Y	Y	Y	Y	N/A	N/A

Sergienko et al. 2015 (Sergienko and Nikonenko 2015)	Y	Y	Y	Y	Y	N	N	N/A	Y	Y	Y	Y	N/A	N/A

Castagno et al. 2017 (Castagno et al. 2017)	Y	Y	Y	Y	Y	N	N	N/A	Y	Y	Y	Y	N/A	N/A

Hashemi et al. 2018 (Hashemi et al. 2018)	Y	Y	Y	N	N	N	N	N/A	Y	Y	Y	Y	N/A	N/A

Benzoni et al. 2007 (Benzoni and Rosenfield 2012)	Y	Y	Y	Y	Y	N	N	N/A	Y	Y	Y	Y	N/A	N/A


**Question key:** Quality assessment tool for cross-sectional and observational studies: Q1 = ‘Was the research question or objective in this paper clearly stated?’; Q2 = ‘Was the study population specified and defined?’; Q3 = ‘Was the participation rate of eligible persons at least 50%’; Q4 = ‘Were all the subjects selected or recruited from the same or similar populations (including the same time period)? Were inclusion and exclusion criteria for being in the study prespecified and applied uniformly to all participants?’; Q5 = ‘Was a sample size justification, power description, or variance and effect estimates provided?’; Q6 = ‘For the analyses in this paper, were the exposure(s) of interest measured prior to the outcome(s) being measured?’; Q7 = ‘Was the timeframe sufficient so that one could reasonably expect to see an association between exposure and outcome if it existed?’; Q8 = ‘For exposures that can vary in amount or level, did the study examine different levels of the exposure as related to the outcome (e.g., categories of exposure, or exposure measured as continuous variable)?’; Q = 9 ‘Were the exposure measures (independent variables) clearly defined, valid, reliable, and implemented consistently across all study participants?’; Q10 = ‘Was the exposure(s) assessed more than once over time?’; Q = 11 ‘Were the outcome measures (dependent variables) clearly defined, valid, reliable, and implemented consistently across all study participants?’; Q12 = ‘Were the outcome assessors blinded to the exposure status of participants?’; Q13 = ‘Was loss to follow-up after baseline 20% or less?’; Q14 = ‘Were key potential confounding variables measured and adjusted statistically for their impact on the relationship between exposure(s) and outcome(s)?’.

## Discussion

In the present study, pooled estimates from sub-group meta-analyses for amplitudes of accommodation (AoA) in a wide range of age groups, from six to 15 years, from five studies with a combined sample of 6,276, were used. All the studies cited in the meta-analysis used the push-up method, in keeping with Hofstetter, to ensure a direct comparison. The results were then compared to the expected AoA for these age groups, based on Hofstetter’s average formula (Hofstetter H 1947). The pooled estimates of AoA demonstrated a statistically significant reduction compared to the expected AoA for ages six, nine, ten, and eleven. The mean difference showed a reduction of 3.4 D, 4.1 D, 4.6 D, and 5.2 D, respectively, for these age groups.

Hofstetter formulae were derived from a predominantly adult population (with only 33 children all above eights of age), and using statistical modelling has allowed for the prediction of paediatric norms. This may be a plausible explanation for the deviations observed from the meta-analyses. The societal implications of children during their developing years having near vision issues whilst learning may result in challenging scholastic performance. The compounded near work of a child’s metaverse in the twenty-first century makes this a pressing issue for clinicians to identify accommodative dysfunction. However, if the normative amplitudes are questionable, this will weaken our clinical prowess to recognise all children with accommodation dysfunction. The meta-analyses pooled estimates attempt to propose more recent norms but also appear to show a gap for more population-based studies for the expected amplitude of accommodation across ethnicities and gender. The findings of the meta-analyses show that Hofstetter’s predicted average for the various age groups overestimates AoA.

The reduction, compared to Hofstetter’s average formula (Hofstetter 1950), agrees with other studies (Iribarren, Fornaciarr & Hung 2001; Castagno et al. 2017). Sterner et al. (Sterner, Gellerstedt & Sjöström 2004) investigated the amplitude of accommodation in children aged six to 10 years using Donder’s push-up method and found lower measurements when compared to Hofstetter’s expected norms. The study revealed monocular values with an average difference (reduction) of 3.5 D. Approximately 51% of children had reduced monocular amplitude, possibly due to accommodative spasm; but it was then postulated that it was highly unlikely that all the participants would have had accommodative spasm to explain the reduced amplitude of accommodation. Castagno et al. (Castagno et al. 2017) also found that the median of AoA in children for the age of 10 years was 15.5 D – also lower than that proposed by Hofstetter’s equation. The authors postulated that there did not appear to be a gradual decline from six to 10 years of age. AoA only decreased after the age of 10, whereas Hofstetter’s equation showed a linear reduction in AoA with age. Their study also found greater variability in the nine- to 12-year age group, with a peak at 10 years. They proposed that the reason for the decline in AoA after ten years of age was because, at higher grades, school children begin to adopt a reading posture with reading distance habits. As they have to interact with long paragraphs, the children develop the ability to use AoA in space, making their amplitude of accommodation more variable. Our study found that the 10 to 11-year-olds did have a lower AoA than Hofstetter’s predicted norms.

Ikaunieks et al. (Ikaunieks et al. 2017) studied the AoA in children aged seven to 15 years and found lower measurements (3.00 D less) for all age groups than the average expected values predicted by Hofstetter’s equation. However, the lower AoA was attributed to the different assessment conditions each investigator adopted to measure AoA. Their study suggests that the task an individual has been doing before a measurement is taken can influence the AoA. For instance, intensive near work may alter the accommodation mechanism in children. Also, because of anatomical differences, younger children read at a closer distance than older children, so perhaps the younger children’s accommodation system will be impacted more than that of older children. Our study noted that younger children in the age group six to nine had lower AoA than Hostetter’s predicted norms.

Hashemi et al. (Hashemi et al. 2018) studied 5444 schoolchildren between the ages of six and 12 years, and other age groups. They also found that the mean measured AoA was less than the predicted mean value calculated with Hofstetter’s formula. They explained that this was possibly due to the inability of the children to understand the meaning of sustained blur, as the endpoint of the push-up-to-blur technique. The study also revealed that their measured AoA was between Hofstetter’s calculated mean and minimum values. This agreed with the standard mean differences observed in our study. However, Hashemi et al. (Hashemi et al. 2018) suggested that Hofstetter’s equation may not be accurate in the study population and proposed that ethnicity be factored into Hofstetter’s equations. The study proposed an amended equation for today’s population: i.e. 16.59 – 0.23 × age. They also raised puberty as another contributing factor in the differences observed.

Other studies (Benzoni and Rosenfield, 2012; Sergienko and Nikonenko 2015) which also agreed with our findings of reduced AoA in comparison to Hofstetter’s expected AoA postulated that the measured AoA in young children might be reduced if the size of the object does not correspond with the patient’s visual acuity. Further to this, the fact that Hofstetter’s equation was extrapolated from Donder’s and Duane’s data mainly took into consideration measurements from subjects over 10 years of age, thus suggesting there might be a variation in certain age groups. This may explain the results for the six- and nine-year-old pool estimates obtained in our study.

The study by Ovenseri-Ogbomo et al. (Ovenseri-Ogbomo et al. 2012), included in our meta-analysis, investigated AoA in Nigerian children and disagreed with our study as they reported a higher mean measured AoA than Hofstetter’s predicted AoA. The study highlighted a more rapid rate of decline in AoA with age and suggested possible reasons for differing from Hofstetter, which included age; ethnicity; and technical differences such as the target size, difference in illumination, and lack of understanding of the first or voluntary effort to clear target blur.

The paucity of studies pre-dating 2000 that used the monocular push-up technique to assess AoA in school children makes for a challenging comparison. In 1961, Eames (Eames 1961) used the binocular push-up to compare sub-urban and urban children’s AoA and showed a mean AoA for 191 eight-year-olds to be 13.70D. However, the pooled estimate (monocular) for this age group was insignificant (p > 0.05) for our study and cannot allow for a valid comparison but may be helpful for illustrative purposes.

Woodruff (Woodruff 1987) used modified Sheard’s technique with the minus lens monocular AoA measured at the spectacle plane. Although this is not a direct comparison of measurements to the pool estimates using the push-up technique, it may indicate the average AoA pre-21st century in 1987 for illustrative purposes. Compared to our significant pooled estimates for the six, nine, ten, and 11-year-old groups, there was comparable magnitude for the nine, ten, and 11-year-old groups from our pooled estimates. However, the technique of push-up-to-blur used in our meta-analyses suggests that if the minus-lens technique was substituted for our meta-analyses’ observation, this might be lower in magnitude than shown due to the influence of linear magnification on the push-up technique.

### Recommendations

Our study suggests that, when using Hofstetter’s formula, a caveat should be issued: that the anticipated measurements will be lower than Hofstetter’s predictions. This review may act as a guide until more robust studies can provide more homogenous pooled estimates from the meta-analysis.

To increase the quality and impact of our study, one should consider expanding the search to young adults as Hofstetter’s formula did use adult population as this may act as a fair comparison. Hofstetter’s formula might require modelling to facilitate demographic profiling, by including race, geographic location, and socioeconomic status.

Practitioners should exercise caution in expecting young children to have high amplitudes of accommodation and should use complementary tests to guard against over-diagnosing under-accommodation. To our knowledge, this is the first meta-analysis that compares the pooled estimates of the AoA from various studies with Hofstetter’s standard measurements in school children. However, the results should be reinforced with more studies with larger samples, across more ethnicities and continents, to support the observations of this review.

### Limitations

Hofstetter employed the push-up technique for measuring AoA. Therefore, to facilitate comparisons with Hofstetter’s estimates, only investigations which used the push-up technique for measuring AoA were reviewed in this study. As a result, research that employed other methods to measure the normative AoA was excluded, potentially reducing the number of studies included in this review. The meta-analysis may have been influenced because the studies included in this review had smaller sample sizes. The meta-analysis of the studies of AoA in children, using a forest plot for age groups seven, eight, and 10 years, revealed significant heterogeneity, shown by the high I^2^ value. Because of this significant heterogeneity, the results for these age groups reveal that there is greater inconsistency in those findings. No direct comparison to normative data from studies pre-dating 2,000 was included and as a pilot search revealed difficulty in locating studies of a similar nature. However, this is worth exploring further to strengthen the working hypothesis of our study.

The quantitative approach of using a meta-analysis methodology that relies on quantitative pooled-estimates did not allow for interrogation into the cited studies’ clinical approaches in factoring clinical attributes when obtaining amplitude of accommodation measurements when dealing with a paediatric population. Such as the accuracy of endpoints for ‘sustained blurred’ when obtaining the AoA, especially at closer distances factoring delayed reaction times at closer endpoints. Incorporating these concerns regarding the sample populations of the cited studies in this review may strengthen the accuracy of cited AoA measurements.

## Conclusion

The results of this systematic review and meta-analysis have elucidated the applicability of Hofstetter’s average expected amplitude of accommodation measurements in young school-going children in the post-digital era of the 21st century. The results revealed that Hofstetter’s average formula for predicting normative AoA today may over-estimate for children aged between six and eleven. Further research is required, with suitably designed population-based studies that will allow a comprehensive evaluation of the amplitude of accommodation in school-going children, factoring in ethnicity, refractive status, digital footprint, ergonomics, and urbanisation of communities. However, the reduction in observed amplitudes of accommodation in the cited age groups may lead to the consideration of assessing for a larger lag of accommodation in six, nine, 10, and 11-year-olds with myopia or pre-myopia as part of the surveillance for progression.
